# Evaluation of Facial Asymmetry Determined by Linear Measurements According to Hand Preference and Gender

**DOI:** 10.1007/s00266-025-04857-1

**Published:** 2025-04-28

**Authors:** Cagatay Barut, Eren Ogut, Asli Dogan Sarikaya, Ozdemir Sevinc

**Affiliations:** 1 School of Medicine, Department of Anatomy, Istanbul Medeniyet University, Istanbul, Türkiye; 2 Faculty of Arts and Sciences, Department of Anthropology, Ahi Evran University, Kırşehir, Türkiye; 3 Faculty of Medicine, Department of Anatomy, Sanko University, Gaziantep, Türkiye

**Keywords:** Facial asymmetry, Hand preference, Sex differences, Cerebral lateralization, Anthropometric measurements

## Abstract

**Introduction:**

Facial asymmetry is a commonly observed phenomenon in human anatomy, with individuals displaying subtle variations between the left and right sides of their face. This study aimed to evaluate facial asymmetry in relation to hand preference and gender.

**Material and Methods:**

Anthropometric facial measurements were conducted on 147 female and 150 male volunteers aged 19–42 years. Standard facial photographs taken from the front in the Frankfort horizontal plane were used to digitally perform 16 linear measurements (8 on the right side and 8 on the left side). All measurements were performed using the AutoCAD program. An asymmetry index was calculated using the (*R*−*L*)(*R*+*L*)×100. The significance of differences between means was tested to evaluate the index values according to sex and hand preference.

**Results:**

Based on the comparison of asymmetry index values between 221 right-handed and 76 left-handed individuals, no statistically significant differences were found across all eight indices. Multiple regression analysis further confirmed that there was no significant relationship between the asymmetry index values and either gender or hand preference.

**Conclusion:**

This study contributes important evidence to the field, showing that facial asymmetry is generally independent of gender and hand preference, and that minor asymmetries are a natural part of human anatomy. Unlike earlier studies that implied stronger connections between handedness or sex and asymmetry, this research emphasizes that facial asymmetry is a stable characteristic across diverse groups.

**Level of Evidence IV:**

This journal requires that authors assign a level of evidence to each article. For a full description of these Evidence-Based Medicine ratings, please refer to the Table of Contents or the online Instructions to Authors  www.springer.com/00266.

## Introduction

During early fetal development, cerebral and craniofacial morphogenesis progress in close proximity [1]. Facial asymmetries appear early in human development, and tend to remain stable throughout life. These minor physical anomalies likely resulted from developmental instability during early embryonic development. Facial asymmetries form in the first trimester of gestation, whereas fluctuating asymmetries develop over time. Several studies have reported positive correlations between fluctuations and facial asymmetry [2]. Small discrepancies between the dimensions of the left and right halves of the face generally do not significantly impact esthetic appearance on their own [3–5]. However, conditions such as cleft palate, hemifacial microsomia, and childhood jaw fractures have been linked to more pronounced facial asymmetry. Moreover, variations in size between the right and left facial halves, termed non-pathological or normal asymmetry, are common in the population [6, 7]. These differences, although often subtle, can occasionally disrupt normal dental function or raise esthetic concerns, albeit sometimes only perceptible through meticulous observation [8, 9]. The emergence of asymmetry in the human skull and face has been hypothesized to arise from uneven development in the cerebral hemispheres, reflecting lateralization [10–12]. Previous research has shown that hemispheric dominance can influence various cognitive and perceptual processes, which may extend to esthetic judgments and facial recognition [13]. Several studies have indicated that handedness may correlate with structural brain differences, which could influence facial asymmetry [14, 15]. Both radiographic and photographic methods have been employed to directly and indirectly measure facial asymmetry, respectively. These assessments encompass traditional morphometric analyses, including measurements of distances, areas, angles, and ratios on both sides of the face [4, 10, 11, 16–18]. Clinical imaging technologies, such as advanced 3D digitization, photography, laser scanning, and light scanning techniques, have enabled the precise quantification of facial landmarks to discern asymmetry and associated conditions [3, 19]. Given the close interplay between facial perception and hemispheric dominance, differences in how individuals perceive faces may vary among right, left, or ambidextrous [20]. Previous studies suggest that hemispheric specialization may influence certain perceptual abilities, including facial recognition and emotional processing, which can vary based on handedness [21]. It has been suggested that while hemispheric dominance could impact facial asymmetry perception, the empirical basis remains mixed, thereby motivating our exploration of hand preference and facial asymmetry. Gender may influence perceptual biases when evaluating facial characteristics [22]. Although we observed differences in facial dimensions between genders, these differences did not significantly impact facial asymmetry in our findings, further highlighting the essential role of evaluator gender in studies of this nature. Studies suggest that while hemispheric dominance may impact esthetic perception, the structural measurements of asymmetry, particularly those measured from linear dimensions, are less influenced by perceptual biases and more so by physiological factors [23, 24]. Therefore, our study centers on quantifying facial asymmetry across hand dominance groups without delving into the subjective perception of asymmetry.

In the literature, asymmetry is commonly defined in various ways, often depending on the specific measurements and techniques used. For instance, some studies utilize landmark-based measurements to calculate asymmetry indices, while others may employ three-dimensional imaging techniques [3, 25, 26]. Our study distinguishes itself by focusing specifically on linear measurements obtained from standardized frontal facial photographs, which allows for a systematic analysis of specific anatomical landmarks. This method provides clear, quantifiable data that can facilitate comparisons across different populations and studies.

Furthermore, we believe that our chosen methodology is particularly appropriate for our research objectives for several reasons. First, linear measurements enable precise assessment of facial dimensions and asymmetry, contributing to a more nuanced understanding of how these factors may relate to gender and handedness. Second, the use of a large sample size (297 individuals) enhances the statistical power of our analysis, allowing for more robust conclusions. Finally, our approach aligns with the methodologies used in recent studies that have similarly highlighted the importance of using standardized measurement techniques to improve reliability and validity in facial asymmetry research [27, 28]. Therefore, in the current study, facial asymmetry was evaluated based on participants'hand preferences and gender.

## Material and Methods

### Participants

This study included 147 female and 150 male volunteers aged 19–42 years. Approval was obtained from the ethics committee of University Faculty of Medicine (2004/05), and informed consent was secured from all participants. The study was conducted in alignment with the Declaration of Helsinki guidelines. Participants were selected based on their willingness to participate and their age eligibility (19–42 years). Exclusion criteria included a history of facial trauma, craniofacial surgery, congenital facial anomalies, or any condition that could affect facial symmetry. Individuals who did not provide informed consent or could not cooperate during measurements were also excluded.

### Procedure

**Fig. 1:**
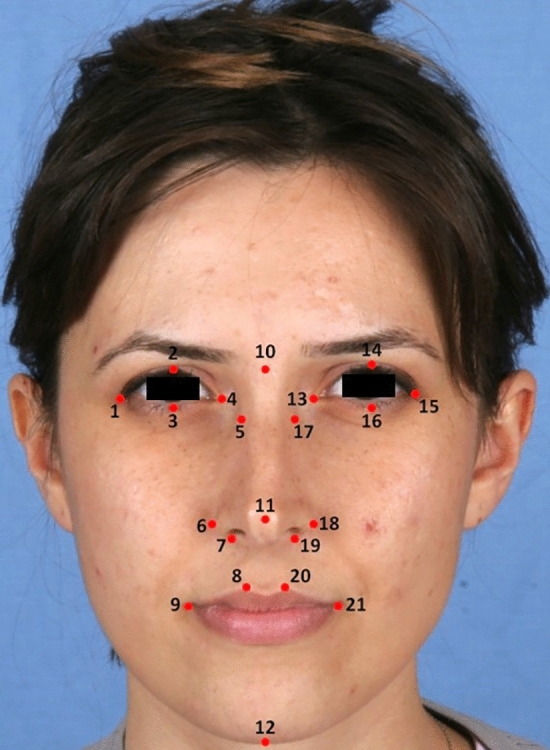
Anthropometric reference points. The numbers in the photograph (1–20) were listed in Table 1

Standardized frontal facial photographs of participants were taken in the Frankfort horizontal plane. The Frankfort horizontal plane is an anatomical reference plane defined by the bilateral alignments of the lowest points on the infraorbital rims and the upper margins of the external ear canal [29, 30]. This plane is commonly used in anthropometric measurements and is significant for ensuring consistent orientation during facial analysis. In our study, we positioned the participants so that their facial photographs were aligned with this plane, which provides a standardized basis for measuring facial asymmetry [31–33]. Specific anatomical landmarks were identified on these photographs, allowing for precise and reproducible linear measurements to assess facial asymmetry. The linear distances between these points were measured to calculate facial asymmetry indices, providing a quantitative assessment of facial asymmetry in relation to hand preference and gender. Landmark descriptions are presented in Table 1. A line connecting the nasion, pronasale, and gnathion established the facial midline, and measurements were taken from each landmark to this midline on both sides of the face. In our research, we defined the midline as a line connecting the nasion, pronasale, and gnathion. This approach aligns with established practices in anthropometric studies, where the midline is typically defined based on key anatomical landmarks that are considered representative of the facial symmetry axis [33]. The distance from each landmark to the midline was measured on both sides of the face. For the palpebra superius and inferius landmarks, the distance between these two points was measured separately on each side of the face. Standard facial photographs taken from the front in the Frankfort horizontal plane were used to digitally perform 16 linear measurements (eight on the right side and eight on the left side) (Fig 1). All measurements were performed using the AutoCAD software (USA). An asymmetry index was calculated using the following formula: [(*R*−*L*)(*R*+*L*)]×100. A difference more than 3% indicates an asymmetry condition regarding the landmarks [34].

**Table 1:** Descriptions of the landmarks used in the study

Numbers	Name of Landmark	Description of landmark
*Midline landmarks*		
10	nasion	The midpoint of the nasofrontal suture
11	pronasale	The most protruded point of the nasal
12	gnathion	The lowest point in the midline on the lower border of the chin
*Bilateral landmarks*		
1, 15	exocanthion	The outer corner of the eye fissure where the eyelids meet
2, 14	palpebrale superius	The highest point on the upper margin of the middle portion of the eyelid
3, 16	palpebrale inferius	The lowest point in the middle of the margin of the lower eyelid
4, 13	endocanthion	The inner corner of the eye fissure where the eyelids meet, not the caruncles
5, 17	maxillofrontale	The anterior lacrimal crest of the maxilla at the frontomaxillary suture
6. 18	alare	The most lateral point on the nasal ala
7, 19	subalare	The point on the lower margin of the base of the nasal ala where the ala disappears into the upper lip skin
8, 20	crista philtre	The point on the crest of the philtrum, the vertical groove in the median portion of the upper lip, just above the vermilion border
9, 21	cheilion	The outer corner of the mouth where the outer edges of the upper and lower vermilions meet

### Selection of Landmarks

The selection of landmarks in our study was based on established anthropometric methods that emphasize key centralized features of the face, which are critical for assessing facial symmetry. Specifically, the landmarks chosen (as shown in Table 1 and Figure 1) correspond to anatomical areas that are traditionally recognized in the field of facial anthropometry [25, 26]. These central landmarks such as the nasion, pronasale, and gnathion were selected for their relevance to overall facial symmetry and their ability to represent a range of dimensional variations across different populations. While it is true that some studies have utilized more lateralized reference points to assess asymmetry [35, 36], our approach is rooted in the understanding that centralized landmarks can effectively capture the essence of facial symmetry. This is particularly relevant given that facial asymmetry often manifests in a general form, which can be evaluated using features that are symmetrically positioned in relation to the midline of the face.

### Data Analysis

Facial asymmetry indices were calculated using Microsoft Excel and statistically analyzed in relation to hand preference and gender. To evaluate the asymmetry-symmetry condition in relation to gender and hand preference the asymmetry index values were analyzed instead of actual linear measurements. All statistical analyses were conducted using SPSS 22.0 (IBM SPSS Software, USA). Descriptive statistics for continuous variables were reported as mean and standard deviation, while categorical variables were described using frequency and percentage. Normality was assessed using the Shapiro-Wilk test. The independent samples t-test was applied for comparing two independent groups with normally distributed variables, and the Mann-Whitney U test was used for non-normally distributed variables. Relation between asymmetry index values and gender and hand preference was further evaluated using multiple regression analysis. A *p*-value of less than 0.05 was considered statistically significant for all comparisons. Hand preference was assessed using the Edinburgh Handedness Inventory [37], and the Geschwind Scores [38] were calculated. The participants were asked 10 questions regarding their hand preferences for writing, drawing, throwing, using various implements like scissors, toothbrush, knife without fork, spoon, striking matches, and jar opening. They were asked to put “+” in the column associated with the hand they used to carry out the activity. Theywere asked to put “++” in the associated column if their preference for one hand was very strong; and to put a “+” in both columns if they were using both hands equally. A “++” in the right column was assigned 10 points, a “+” in the right column 5 points, whereas a “++” in the left column –10 points and a “+” in the left column –5 points. The sum of these points was used to determine the Geschwind Score (GS), as an indicator of the direction and degree of hand preference. Hand preference was evaluated in 5 groups depending on the value of GS, strong right-handers: +80*<*GS*<*+100, weak right-handers: +20*<*GS*<*+75, ambidexters: −15*<*GS*<*+15, weak left-handers: –75*<*GS*<*−20, and strong left-handers: –100*<*GS*<*−80 [38–42]. However, ambidextrous individuals were excluded from the study due to their small number. To evaluate the obtained index values based on sex and hand preference, significance testing was conducted on the difference between the two means. The significance of differences between means was tested to evaluate the index values according to sex and hand preference. Based on the analysis of facial photographs, the following asymmetry indices were measured:

1. Asymmetry Index 1: Distance from the right maxillofrontale to the midline compared to the distance from the left maxillofrontale to the midline.
2. Asymmetry Index 2: Distance from the right alare to the midline compared with the distance from the left alare to the midline.
3. Asymmetry Index 3: Distance from the right subalare to the midline compared with the distance from the left subalare to the midline.
4. Asymmetry Index 4: Distance from the right crista philtri to the midline compared with the distance from the left crista philtri to the midline.
5. Asymmetry Index 5: Distance from the right cheilion to the midline compared to the distance from the left cheilion to the midline.
6. Asymmetry Index 6: Distance from the right endocanthion to the midline compared to the distance from the left endocanthion to the midline.
7. Asymmetry Index 7: Distance from the right exocanthion to the midline compared to the distance from the left exocanthion to the midline.
8. Asymmetry Index 8: Distance between the right palpebra superius and palpebra inferius compared to the distance between the left palpebra superius and palpebra inferius (Table 2)

**Table 2:** Description of measurements on both sides according to the asymmetry index

Name of index	Measurement on the right side	Measurement on the left side
Asymmetry index 1	Right maxillofrontale—midline	Left maxillofrontale—midline
Asymmetry index 2	Right alare—midline	Left alare—midline
Asymmetry index 3	Right subalare—midline	Left subalare—midline
Asymmetry index 4	Right crista philtre—midline	Left crista philtre—midline
Asymmetry index 5	Right cheilion—midline	Left cheilion—midline
Asymmetry index 6	Right endocanthion—midline	Left endocanthion—midline
Asymmetry index 7	Right exocanthion—midline	Left exocanthion—midline
Asymmetry index 8	Right palpebra superius—palpebra inferius	Left palpebra superius—palpebra inferius

## Results

**Fig. 2:**
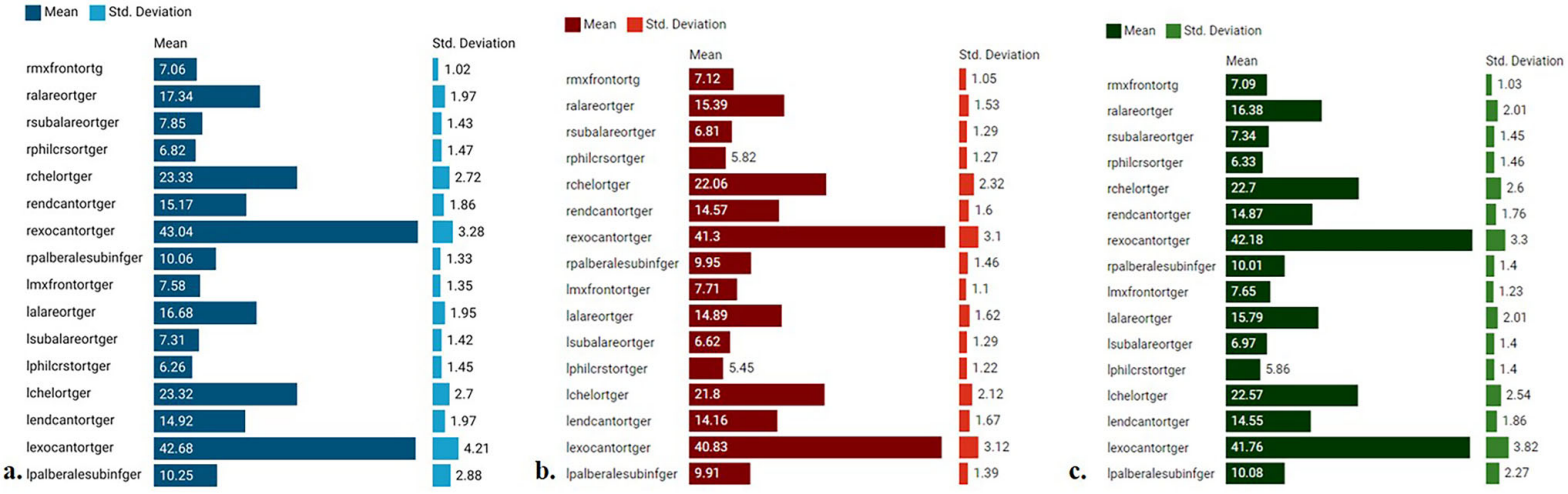
Descriptive statistics of parameter **a.** Descriptive statistics of the parameters in males **b.** descriptive statistics of the parameters in females **c.** descriptive statistics of the parameters in total. The following parameters were measured on both sides as shown in Figure 2: maxillofrontale, alare, subalare, crista philtri, cheilion, endocanthion, exocanthion, palpebrale superius, and palpebrale inferius. r: right, l: left

Facial photographs of 297 individuals (150 males and 147 females) were analyzed. Degree of hand preference was not taken into account as there were not enough subjects in some subgroups. Among these individuals, 221 were right-handed and 76 were left-handed (Table 3). The descriptive statistics of the measured parameters are shown in Fig. 2. Statistical analysis revealed no significant differences in asymmetry indices between the right and left measurements (*p* > 0.05). This study measured various facial parameters in 297 individuals, revealing that males generally had larger measurements than females across all landmarks. Notably, the right alare to midline and right cheilion to midline distances showed the largest differences between sexes. These findings suggest that while both male and female facial structures are asymmetrical, males tend to have slightly larger dimensions, indicating a potential influence of sex on facial asymmetry measurements. However, based on the comparison of asymmetry index values between the 150 males and 147 females, no statistically significant differences were observed across all eight indices (*p* > 0.05). This suggests that there is no significant gender-based variation in facial asymmetry, as measured by these indices (Table 4). Based on the comparison of asymmetry index values between 221 right-handed and 76 left-handed individuals, no statistically significant differences were found across all eight indices (*p* > 0.05). This indicates that hand preference does not significantly influence facial asymmetry as measured by these indices (Table 5). Multiple regression analysis further confirmed that there was no significant relationship between the asymmetry index values and either gender or hand preference. These findings suggest that hand preference and sex do not have a substantial impact on facial asymmetry (Table 6). This analysis confirmed that neither gender nor hand preference had a statistically significant impact on the asymmetry index values (all *p* >0.05), reinforcing that these factors do not substantially influence facial asymmetry.

**Table 3:** Distribution of hand preference of participants in relation to gender

Degree of Hand Preference	Males		Females		Total	
	n	%	n	%	n	%
Strong right-handers	16	10.7	31	21.1	47	15.8
Weak right-handers	82	54.7	92	62.6	174	58.6
Weak left-handers	36	24.0	14	9.5	50	16.8
Strong left-handers	16	10.7	10	6.8	26	8.8
Total	150	100.0	147	100.0	297	100.0

	Males		Females		Total	
Hand preference	n	%	n	%	n	%
Right-handers	98	65.3	123	83.7	221	74.4
Left-handers	52	34.7	24	16.3	76	25.6
Total	150	100.0	147	100.0	297	100.0

**Table 4:** Comparison of asymmetry index values according to gender

	Male (*N*= 150)		Female (*N*= 147)				
	Mean	SD	Mean	SD	*t*	df	*p*
Asymmetry index 1 (%)	− 3.306	10.193	− 3.987	9.428	0.597	295	0.551
Asymmetry index 2 (%)	1.941	6.932	1.725	5.914	0.290	289.524	0.772
Asymmetry index 3 (%)	3.650	10.193	1.485	9.381	1.904	295	0.058
Asymmetry index 4 (%)	4.275	16.121	3.307	14.993	0.536	295	0.593
Asymmetry index 5 (%)	− 0.002	5.415	0.551	5.012	− 0.912	295	0.363
Asymmetry index 6 (%)	0.851	8.722	1.457	7.057	− 0.659	284.845	0.510
Asymmetry index 7 (%)	0.512	4.957	0.587	2.832	− 0.161	237.837	0.873
Asymmetry index 8 (%)	− 0.301	5.799	0.143	2.742	− 0.839	295	0.402

Independent samples test

**Table 5:** Comparison of asymmetry index values according to hand preference

	Right hander (*N*=221)		Left hander (*N*=76)				
	Mean	SD	Mean	SD	t	df	p
Asymmetry index 1 (%)	− 3.036	9.843	− 5.409	9.565	1.826	295	0.069
Asymmetry index 2 (%)	2.035	6.421	1.251	6.493	0.916	295	0.361
Asymmetry index 3 (%)	2.321	10.054	3.325	9.222	− 0.766	295	0.444
Asymmetry index 4 (%)	4.494	15.395	1.766	15.936	1.321	295	0.188
Asymmetry index 5 (%)	0.246	5.253	0.346	5.151	− 0.144	295	0.886
Asymmetry index 6 (%)	1.561	7.942	− 0.042	7.841	1.523	295	0.129
Asymmetry index 7 (%)	0.638	4.244	0.290	3.392	0.647	295	0.518
Asymmetry index 8 (%)	0.178	2.876	− 0.835	7.523	1.681	295	0.094

Independent samples test

**Table 6:** Multiple regression analysis of asymmetry index values and gender and hand preference

Dependent Parameter	SumSq	df	R^2^	F	p	Independent Variable	Beta	t	p
Asymmetry index 1	415.241	2	0.015	2.174	0.116	Gender	− 0.60	− 1.007	0.315
						Hand preference	− 0.118	− 1.997	0.047
Asymmetry index 2	44.869	2	0.004	0.540	0.584	Gender	− 0.029	− 0.493	0.623
						Hand preference	− 0.059	− 0.998	0.319
Asymmetry index 3	361.704	2	0.013	1.878	0.155	Gender	− 0.105	− 1.779	0.076
						Hand preference	0.022	0.378	0.706
Asymmetry index 4	588.444	2	0.008	1.218	0.297	Gender	− 0.40	− 0.833	0.406
						Hand preference	− 0.087	− 1.466	0.144
Asymmetry index 5	25.866	2	0.003	0.473	0.623	Gender	0.057	0.962	0.337
						Hand preference	0.020	0.343	0.732
Asymmetry index 6	152.912	2	0.008	1.216	0.298	Gender	0.021	0.347	0.726
						Hand preference	− 0.084	− 1.414	0.159
Asymmetry index 7	6.860	2	0.001	0.209	0.812	Gender	0.001	0.024	0.981
						Hand preference	− 0.037	− 0.626	0.531
Asymmetry index 8	63.208	2	0.010	1.533	0.218	Gender	0.030	0.500	0.618
						Hand preference	− 0.091	− 1.536	0.126

The descriptive statistics of the facial measurements for 150 males and 147 females indicated notable differences in specific parameters. Males generally exhibited larger mean values compared to females, particularly in measurements such as right alare to midline (17.34 mm vs. 15.39 mm) and right cheilion to midline (23.33 mm vs. 22.06 mm). These differences are consistent across various landmarks, suggesting a potential influence of gender on facial dimensions. However, main focus of the current study is asymmetry index values which reflect symmetry and asymmetry conditon of the face, and thus individual linear measurements were not statistically analyzed. These parameters are presented in Table 7.

**Table 7:** Descriptive statistics of measured parameters

Parameter	Male (*N*=150)		Female (*N*=147)		Total (*N*=297)	
	Mean	SD	Mean	SD	Mean	SD
Right maxillofrontale—midline (mm)	7.06	1.02	7.12	1.05	7.09	1.03
Right alare—midline (mm)	17.34	1.97	15.39	1.53	16.38	2.01
Right subalare—midline (mm)	7.85	1.43	6.81	1.29	7.34	1.45
Right crista philtre—midline (mm)	6.82	1.47	5.82	1.27	6.33	1.46
Right cheilion—midline (mm)	23.33	2.72	22.06	2.31	22.70	2.60
Right endocanthion—midline (mm)	15.17	1.86	14.57	1.6	14.87	1.76
Right exocanthion—midline (mm)	43.04	3.28	41.30	3.10	42.18	3.30
Right palpebra superius—palpebra inferius (mm)	10.06	1.33	9.95	1.46	10.01	1.40
Left maxillofrontale—midline (mm)	7.58	1.35	7.71	1.10	7.65	1.23
Left alare—midline (mm)	16.68	1.95	14.89	1.62	15.79	2.01
Left subalare—midline (mm)	7.31	1.42	6.62	1.29	6.97	1.40
Left crista philtre—midline (mm)	6.26	1.45	5.45	1.22	5.86	1.40
Left cheilion—midline (mm)	23.32	2.70	21.80	2.12	22.57	2.54
Left endocanthion—midline (mm)	14.92	1.97	14.16	1.67	14.55	1.86
Left exocanthion—midline (mm)	42.68	4.21	40.83	3.12	41.76	3.82
Left palpebra superius—palpebra inferius (mm)	10.25	2.88	9.91	1.39	10.08	2.27

## Discussion

The current study revealed that both male and female faces exhibit asymmetry, with males generally showing larger measurements across various facial landmarks. However, there were no statistically significant differences in the facial asymmetry indices between the sexes or between right- and left-handed individuals. These results suggest that sex and hand preference do not significantly influence facial asymmetry. However, the utilization of asymmetry index values obtained from measured linear lengths is a distinct methodology compared with the existing literature. The discrepancy with the literature results is attributed to potential methodological differences.

### Gender

Smith et al. reported that male and female faces exhibited asymmetry in opposing directions: males tended to have a greater left-sided facial area compared to the right, while females tended to exhibit a larger right-sided area relative to the left [8]. Additionally, the disparity in facial asymmetry between sexes is attributed to differential muscular development on each side of the face [8]. It was reported that females exhibited a higher number of significantly asymmetric linear distances between the two halves of the face than males. Additionally, the left side of the face tends to be more dominant in both males and females [43]. Facial and body asymmetries may indicate various underlying factors. While facial symmetry is often associated with facial attractiveness, faces typically exhibit asymmetry, with one side frequently displaying greater size or prominence than the other [44]. Two databases of facial images from Stirling and St Andrews Universities, comprising 180 and 122 faces, respectively, along with a third set of 62 faces collected at Abertay University, were used to investigate Smith's findings. Overall, no significant sex effect on facial area asymmetry was detected. However, the St Andrews’ sample demonstrated a pattern similar to Smith's observations, where females exhibited a significantly larger mean area on the right hemiface and males on the left hemiface. Furthermore, among Abertay faces, handedness significantly influenced facial asymmetry, with right-handers displaying a larger left side of the face. These results partially support Smith's findings but also indicate that detecting such asymmetries may depend on unidentified factors inherent in certain methods of image collection [44]. In our study, we have identified a lack of significant differences in facial asymmetry related to gender and hand preference, feature-dependent definition of asymmetry. This approach may not align with the methodologies employed in previous research, which often defined facedness in terms of the relative sizes of the two hemifaces, as noted in studies by [8] and [44]. These methodologies offer a more comprehensive view of facial asymmetry by considering overall facial proportions. Additionally, recent study by [45] employed two complementary 3D techniques, illustrating the potential for detecting differences in asymmetry Some studies focus on centralized, feature-dependent measurements, while others may evaluate asymmetry based on the relative sizes of hemifaces or utilize advanced 3D modeling techniques. Resent studies employed both landmark and polygonal surface analysis using color-coded maps, allowing for a more nuanced understanding of facial asymmetry and its potential gender-related differences [45–47]. By integrating such contemporary methodologies, future investigations can offer deeper insights and potentially reveal subtle differences that may have been overlooked in traditional 2D approaches.

Factors other than symmetry have also been reported to play a role in assessing phenotypic conditions. One such indicator is facial masculinity, characterized by prominent cheekbones and a relatively elongated lower face, which has been associated with attractiveness in both full-face and half-face evaluations [9]. A previous study reported that the mean faces of both groups exhibited significant asymmetry in shape, indicating notable differences between the two sides of the face; however, no differences in size were reported [3]. Patients of both sexes exhibited a complex 3D shape change pattern characterized by an elongated lower mid-facial height, shortened upper mid-facial height, a posteriorly located nasion, and a broader posterior face. Additionally, there was sex-specific rotation of the midface, with the base of the nose positioned more anteriorly in female patients and more posteriorly in male patients. Notably, sex-specific asymmetries were observed: in males, controls displayed significant directional asymmetry, whereas patients exhibited reduced directional asymmetry. Conversely, in females, controls showed minimal directional asymmetry, whereas patients displayed pronounced directional asymmetry [1]. A previous study reported that right-left differences in the hand-shape index and palmar length/width values of strong left-handers, weak left-handers, and ambidextrous individuals were statistically significant according to sex [40]. In the current study, there were no significant differences in most asymmetry index values between males and females. However, asymmetry index 3 showed a near-significant difference (*p *= 0.058), with males having a higher mean value than females. Specific facial measurements indicated that males generally had larger measurements than females, particularly in areas such as the maxillofrontale-midline, alare-midline, and cheilion-midline on both sides of the face. These findings align with the literature, suggesting sex-based differences in facial asymmetry. Males and females exhibit distinct patterns of facial asymmetry, which may be influenced by factors such as hormonal levels and muscular development. These differences were observable in specific facial measurements, supporting the notion that sex plays a significant role in facial asymmetry. The larger dimensions observed in males compared to females, particularly in parameters such as right alare to midline and right cheilion to midline, highlight the potential influence of gender on facial morphology. These indices may be useful for further studies aimed at exploring facial asymmetry in different populations. They can serve as baseline measurements for understanding normative variations and could be applied in clinical settings to assess individual cases of asymmetry related to various conditions.

### Age

It has been reported that asymmetry does not correlate with twin zygosity, adolescence, or age [10]. In a study examining the effects of sex and age on 3D soft tissue facial asymmetry, no age-related differences were observed in both absolute and percentage distances from symmetry. The maximum normal asymmetry was marginally higher in females than in males of similar age groups, with the greatest values typically seen in adolescents of each sex. The tragion, gonion, and zygion emerged as the most asymmetric landmarks across all cohorts, while the endocanthion exhibited the least asymmetry. Mild soft tissue facial asymmetry was noted in healthy subjects. These findings suggest that identifying patients with borderline asymmetry may benefit from assessing maximum normal asymmetry [4]. It was reported that asymmetry decreases with age until the age of 11 years. This was followed by an increase in facial asymmetry, which peaked at 13 years in males and 14 years in females. From age 15, a reduction in facial asymmetry was maintained until the age of 18, which may have been explained by the interaction between rapid growth and a high metabolic rate in children or increased secretion of sex steroids during adolescence [48]. In one study, more pronounced asymmetry compared to other age groups was observed among males aged 20–40 years. Furthermore, significant sexual dimorphism in directional asymmetry declines rapidly after middle age, likely due to age-related changes in facial structure [49]. Facial morphology is significantly influenced by androgens, suggesting a role of sex hormones in facial asymmetry. This contributes to the observation that facial asymmetry tends to converge between sexes with the onset of declining sex hormone levels after the age of 40 [50]. It has been suggested that facial asymmetry is strongly dependent on age, which increases over time [51, 52]. This could also be attributed to delayed ossification processes and ethnic variation [53]. Asymmetries can occur during any period of development and are particularly high in children during periods of rapid growth [2]. Possible causes include asymmetric growth leading to suture displacement, sutural synostosis of the skull on the affected side, or ethnic predisposition [54–56]. This highlights the importance of considering broader age ranges when studying the impact of age on facial asymmetry, as significant differences may only become apparent in younger or older populations.

### Hand Prefrence

Most individuals exhibit a hand preference in various tasks, identifying themselves as either right- or left-handed. Handedness, or hand dominance, is the most recognized and extensively studied form of human behavioral asymmetry. However, in many bimanual tasks, each hand often assumes a specific role even though the preferred hand typically serves as the dominant hand [57]. In a study using fMRI to examine the frontal cortex, 96% of right-handed participants exhibited fMRI changes lateralized to the left hemisphere, with the remaining 4% showing bilateral activation patterns. Among the left-handed individuals, 76% demonstrated left hemisphere lateralization, 14% bilateral activation, and 10% right hemisphere lateralization. However, right hemisphere dominance was minimal; only one left-handed participant (2%) exhibited complete right hemisphere lateralization. During silent word generation, both the right- and left-handed groups exhibited left hemisphere lateralization, although right hemisphere involvement was dominant in typical left-handed individuals. Exclusive activation of the right hemisphere in the frontal lobe is rare [58]. There is a lack of consensus regarding the degree, side, and spatial localization of facial asymmetry. Significant facial asymmetry has been consistently demonstrated in various studies, even among esthetically pleasing faces [59]. However, there is no consensus regarding the dominant side [3]. Some authors have suggested that the hemispheres of ambidextrous and left-handed individuals'brains were nearly symmetric and that the equal use of hands in ambidextrous subjects was considered a marker indicating a failure to establish cerebral dominance in either hemisphere [60]. It has been reported that within brain functioning, processes of face perception are predominantly lateralized and strongly associated with the right hemisphere. However, the external manifestation of hemispheric dominance, indicated by contralateral handedness or the dominance of bilateral pairs of structures in the body, varies among subjects [20]. It has been reported that the occurrence of left-facedness among right-handed individuals has been interpreted as suggesting dominance of the right cerebral hemisphere in the control of facial expression of emotions and movement of the facial musculature [61–63]. Including facedness data from left-handed subjects would provide a clearer investigation into whether the hemisphere responsible for facial expression differs from the dominant hemisphere for other lateralized motor functions [64]. It has been reported that right-handers exhibit a significant left-faced orientation, while left-handers tend to show left-faced tendencies. This suggests that facial asymmetry during emotional expression may not be influenced by handedness but rather by the dominance of the right hemisphere. However, these observations may be influenced more by the method of handedness assessment, such as self-reporting, than by the underlying distribution [64]. Therefore, it has been suggested that variations in facial shape and asymmetry may reflect variations in adult brain function [1]. It has been noted that differences in hand morphology exist between sexes, possibly influenced by varying prenatal exposure to testosterone and estrogen, in addition to genetic factors [65]. Moreover it has been indicated that cultural differences among study groups could account for inconsistencies in hand and foot preference [39]. It has been reported that left-handers show inconsistent facial asymmetry patterns, often exhibiting larger facial areas on the right than on the left. Sex differences were particularly notable among left-handed participants. These findings suggest that asymmetric development in certain brain regions may contribute to the development of asymmetry in the facial regions [66]. One study reported that right-handers exhibited a larger left craniofacial region, whereas left-handers showed a larger right craniofacial region. These findings support the hypothesis that hand preference may correlate with craniofacial asymmetries, potentially extending to aural asymmetries [67]. In a study evaluating functional laterality (hand preference) and mandibular asymmetry in normodivergent patients using panoramic radiographic images, it was found that hand-use preference was not associated with face and condylar, ramal, or total mandibular asymmetry [68]. This study’s findings align with existing literature that suggests handedness may not be directly correlated with craniofacial or facial asymmetry, emphasizing that possible biological and environmental factors likely contribute to asymmetry in facial and craniofacial features. Handedness alone may not serve as a predictor for such asymmetries, and future studies may benefit from integrating broader markers of asymmetry and exploring cultural or developmental factors that contribute to these patterns.

### Clinical Significance

During the preoperative evaluation of facial surgery and orthodontic procedures, facial asymmetry should be carefully assessed and may only become apparent through detailed morphometric analysis [43]. The degree of asymmetry was notably higher in unhealthy individuals, where irregular development of skeletal, dental, and soft tissues could variably contribute to clinically observable imbalances [4, 18]. Studies have shown that asymmetries are significantly more pronounced in patients with malocclusion, highlighting the importance of addressing these factors during diagnosis and treatment planning for patients with facial asymmetries [69]. Research on directional facial asymmetry, including age-related changes, plays a critical role in assessing the treatment of craniofacial malformations and trauma in orthodontics, facial surgery, and forensic sciences [49]. This emphasizes the necessity for a comprehensive diagnostic workup incorporating medical and dental histories, clinical examinations, study models, and photographic and radiographic records. Such an approach is essential for identifying craniofacial defects and preventing significant functional and esthetic complications [70]. The findings of current study on facial asymmetry as a stable anatomical feature align with the growing use of digital measurement systems in clinical practice. Just as versatile digital tools enhance objective assessments in plastic surgery and wound healing prediction, they could also be instrumental in analyzing and monitoring facial asymmetries over time [71]. The integration of technology-driven assessments such as 3D imaging, AI-based analysis, and predictive scoring systems like the modified-TIME-H could provide more standardized, reproducible, and clinically relevant data for both esthetic and reconstructive procedures [72]. As technology evolves, advanced imaging and AI-driven systems could provide real-time data analysis, predicting outcomes and offering more personalized treatment plans. Additionally, Patient-Reported Outcome Measures (PROMs) like SCAR-Q, BREAST-Q, and BODY-Q are essential tools in assessing patient satisfaction and functional results, particularly in esthetic and reconstructive surgeries. These PROMs address both physical and psychological recovery, and their ongoing validation is expected to further enhance patient-centered care in plastic surgery [72–75]. While factors like handedness may contribute to certain craniofacial patterns, they do not serve as definitive predictors of facial asymmetry. A multifactorial approach, considering both clinical and functional perspectives, is essential in accurately diagnosing and planning individualized treatments for facial asymmetry. Recognizing facial asymmetry as a stable trait helps clinicians differentiate between natural variations and pathological cases, reducing unnecessary medical concern and guiding more precise surgical planning. This understanding also improves patient education, alleviating anxiety in esthetic medicine and enabling more personalized orthodontic and prosthetic treatments.

### Future Directions

Cross-cultural studies should be conducted to determine whether patterns of facial asymmetry differ among diverse populations. These studies could provide insights into the influence of ethnicity and cultural practices on craniofacial development. Potential correlations between facial asymmetry and behavioral traits, including cognitive function and personality, should also be explored to understand the broader implications of craniofacial asymmetry. Additionally, longitudinal studies should be conducted to track changes in facial asymmetry over time within the same individual, which could help identify critical periods of asymmetrical development. With advancements in 3D imaging, AI-driven analysis, and objective measurement tools, future studies could integrate technology-driven assessments to further validate and refine our understanding of facial asymmetry. These developments may contribute to a more comprehensive and objective approach to asymmetry analysis, bridging the gap between anatomical findings and patient-centered outcomes in plastic surgery.

### Limitations

The limited sample size and regional focus may restrict the broader applicability of the findings to global populations with diverse ethnic backgrounds and environmental influences.

## Conclusion

This study contributes to the literature by providing essential evidence that facial asymmetry is largely independent of gender and hand preference, reinforcing the understanding that minor asymmetries are a normative aspect of human morphology. Unlike previous studies that may have suggested more pronounced differences based on these factors, our findings highlight the necessity of comprehensive morphometric analyses, indicating that variations in facial dimensions do not translate into significant asymmetry indices across different individuals. Additionally facial asymmetry may underlie structural differences related to gender and handedness which are less influential in this context.

## Data Availability

Not applicable.
